# True Intra-Articular Lipoma of the Knee: A Case Report

**DOI:** 10.7759/cureus.95583

**Published:** 2025-10-28

**Authors:** Hugo Vaz dos Santos, Raquel Costa, André Castanheira, Luís Barbosa, Luís Machado

**Affiliations:** 1 Orthopedics and Trauma, Hospital Beatriz Ângelo, Loures, PRT; 2 Orthopedic Surgery, Hospital Beatriz Ângelo, Loures, PRT; 3 Orthopedic Surgery, Hospital da Luz, Lisbon, PRT

**Keywords:** case report, intra-articular lipoma, lipoma, mri, open surgery, synovial lipoma

## Abstract

Although lipomas are the most common benign soft tissue tumor, very few cases of true intra-articular lipomas have been described. They are usually small in size and located at the knee. The differential diagnosis of an intra-articular lipomatous mass is made between lipoma arborescens and synovial lipoma. We report a case of a true intra-articular lipoma of the knee in a 60-year-old woman who presented with knee pain and a large anteromedial mass. The magnetic resonance imaging (MRI) confirmed the presence of a well-delineated fat-containing mass, and the lipoma was excised by a standard medial arthrotomy. After two years, the patient reported no limitation in her daily life.

## Introduction

Lipomas are the most common benign soft tissue tumors, accounting for nearly half of all benign mesenchymal neoplasms [1]. They usually occur in subcutaneous or intramuscular locations; however, intra-articular presentation is exceedingly rare. Among these, the knee joint is most frequently affected, although cases have also been described in the hip, lumbar spine, and tarsometatarsal joints [2-4].

When an intra-articular fatty mass is identified, the main diagnostic consideration lies between a true intra-articular synovial lipoma and lipoma arborescens. Clinically, lipoma arborescens is more frequent, typically presenting with gradual onset of swelling and effusion. It is thought to represent a nonspecific reactive fatty proliferation of the synovium secondary to chronic traumatic or inflammatory stimuli, rather than a true neoplastic process [5]. In contrast, true intra-articular lipomas present as slowly enlarging, well-circumscribed masses that may cause restricted motion or, less commonly, mechanical symptoms such as patellar instability or acute pain due to strangulation [6-8]. Macroscopically, lipoma arborescens displays a characteristic villous or “tree-like” architecture with multiple fatty projections, whereas true lipomas are solitary, encapsulated, and smooth-surfaced. On magnetic resonance imaging (MRI), both lesions share the typical features of fat-containing tissue-high signal intensity on T1- and T2-weighted sequences with complete suppression on fat-saturated images but differ in morphology: lipoma arborescens shows frond-like synovial proliferation and joint effusion, while true lipomas appear homogeneous and well-encapsulated. Histologically, lipoma arborescens consists of mature adipose tissue replacing the subsynovial connective tissue, lined by hypertrophic synovium; in contrast, true lipomas are composed of mature adipocytes enclosed by a thin fibrous capsule without synovial villous architecture [5,9].

Because of their rarity, true intra-articular lipomas can pose a diagnostic challenge, as their presentation may mimic other intra-articular lesions. MRI remains the modality of choice for characterization [10]. Surgical excision is curative, with either open or arthroscopic approaches depending on the lesion’s size and location [11].

We report the case of a large true intra-articular lipoma of the knee in a 60-year-old woman, emphasizing its clinical presentation, radiologic findings, surgical management, and a brief review of the literature. The aim of this report is to highlight the distinguishing features and management considerations of this rare entity.

## Case presentation

A 60-year-old woman presented to our outpatient clinic with a six-year history of progressive left knee pain and a “permanent swelling” that gradually increased in size. She related the onset of her symptoms to a fall six years earlier. The swelling had been palpable for several years but had recently become more prominent, with increasing discomfort and mechanical limitation. During the months preceding her appointment, her symptoms worsened, limiting her daily activities, particularly walking medium distances and climbing stairs. She also reported morning stiffness and difficulty bending the knee, prompting medical evaluation. Apart from being overweight and leading a sedentary lifestyle, her medical history was unremarkable. She denied previous surgeries, and her family history was non-contributory.

Physical examination revealed an anteromedial soft tissue mass that was palpable and tender to touch, grossly measuring approximately 8 × 6 cm. There was no erythema or other inflammatory signs around the knee. Pain was elicited on palpation of the medial joint line. A mild joint effusion was confirmed by a positive patellar tap test. The range of motion was limited to 100° of flexion due to pain and mass effect, while extension was preserved. No locking, catching, or signs of meniscal or ligamentous injury were observed, and overall alignment and joint stability were normal. She had no history or clinical evidence of patellar instability. In summary, the patient exhibited a slow-growing, palpable, well-defined intra-articular mass associated with mechanical limitation and absence of inflammatory signs.

**Figure 1:**
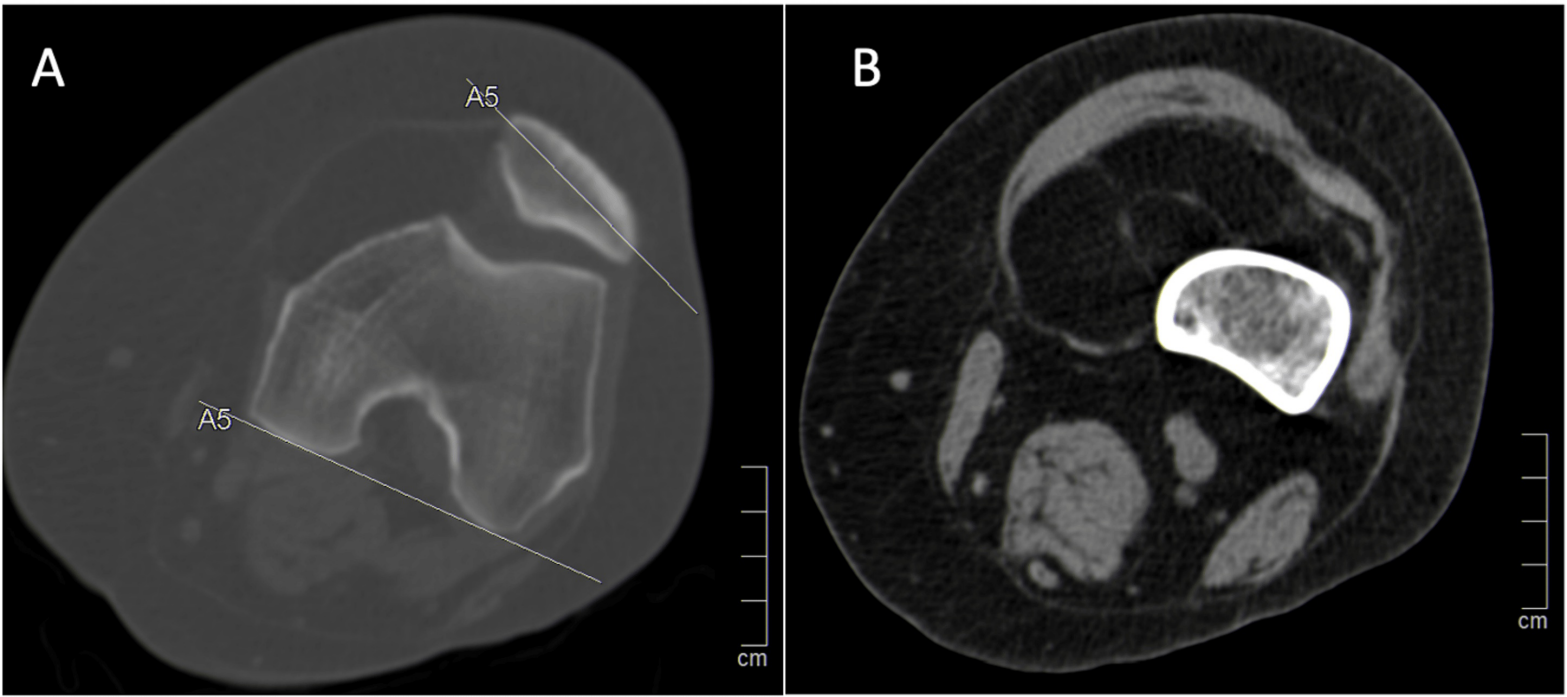
CT images of the left knee (A) Axial view showing a well-defined intra-articular lesion, with a slight patellar tilt of 22º due to mass effect. (B) Axial view showing the homogeneous fat attenuation mass with thin septations.

Her biochemical and serologic examinations, including C-reactive protein, erythrocyte sedimentation rate, and rheumatoid factor, were normal. A computed tomography (CT) scan was first performed, showing an intra-articular mass starting to invade the space between the femoral condyles and the articular facet of the patella, dislocating it laterally, with a slight tilt of the patella (Figure 1).

**Figure 2:**
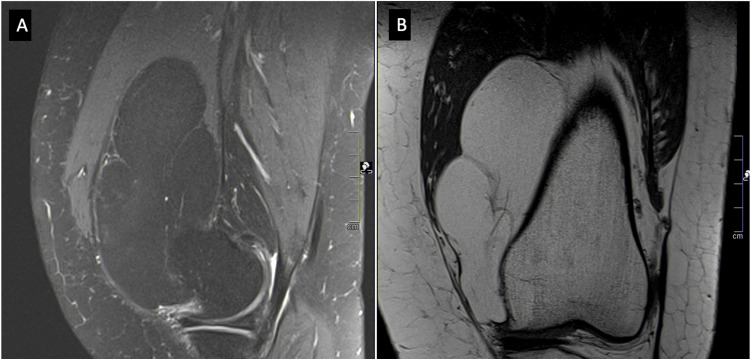
MRI images of the left knee (A) Sagittal PD FS MRI sequence (0.6 mm thickness) showing a hypointense signal, confirming the presence of intralesional fat. (B) Coronal T1-weighted MRI showing a homogeneous, well-defined hyperintense lesion, measuring 11 × 5 × 5 cm, with thin septations. PD, proton density; FS, fat suppression; MRI, magnetic resonance imaging

The MRI revealed a homogeneous, well-defined mass, measuring 11 x 5 x 5 cm, with thin septations located at the anteromedial aspect of the knee. The tumor showed a hyperintense signal on the coronal T1-weighted and proton density (PD)-weighted sequences, losing its signal when fat-suppression (FS) technique was applied to the PD sequence (Figure 2).

**Figure 3:**
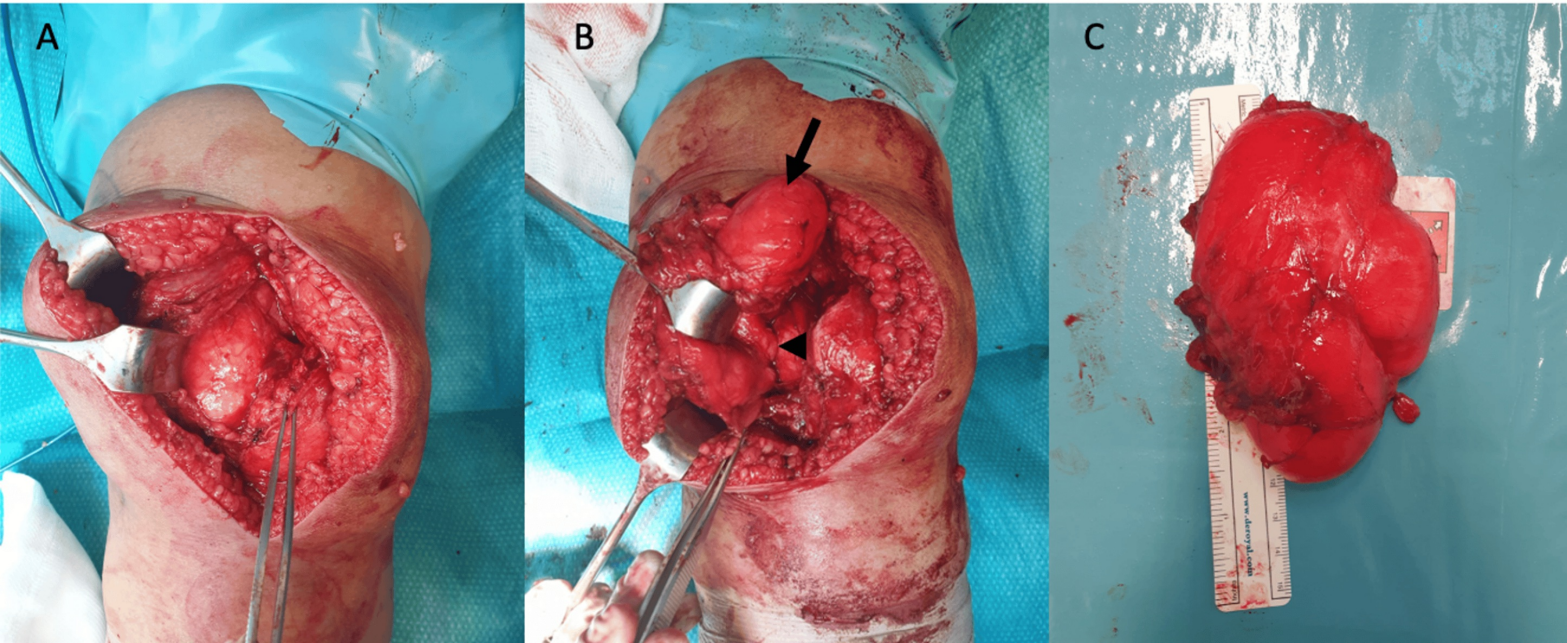
Intraoperative images (A) Surgical excision of the mass using an open medial parapatellar approach. (B) The lipoma appears well encapsulated, with clear margins (arrow) and a vascular pedicle (arrowhead). (C) Excised intra-articular lipoma measuring 11 × 6 × 5 cm.

Due to the size of the mass, the excision was made by a standard open medial parapatellar approach. The mass, measuring 11 x 6 x 5 cm, seemed to arise from the suprapatellar pouch growing upward and toward the medial aspect of the knee and distal third of the thigh. Macroscopically, a capsule could be seen, and the mass was well defined, with a bright yellow/pink color. It had a vascular stalk, with no signs of strangulation (Figure 3).

**Figure 4:**
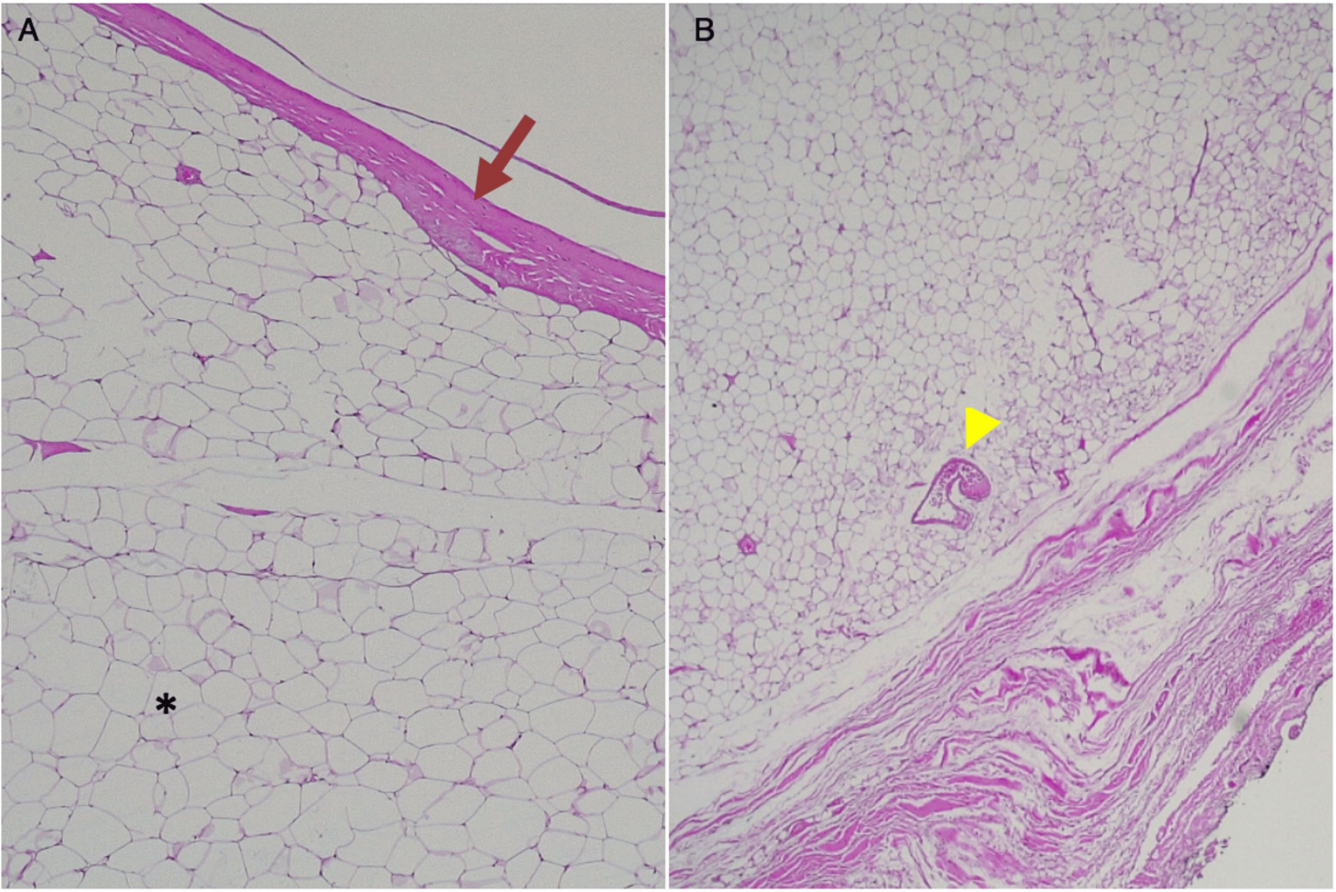
Histologic appearance (A) Histologic appearance of the mass demonstrating mature adipocytes (asterisk) encapsulated by a thin fibrous layer (arrow). (B) Vessels are also evident (arrowhead), hematoxylin and eosin (A, x40; B, x25).

Histologic examination showed a homogenous tumor composed of mature adipocytes, without atypia, surrounded by a dense fibrous capsule, and some blood vessels were also visible. There were no signs of inflammatory infiltrate (Figure 4).

The mass was compatible with an intra-articular synovial lipoma, and the final diagnosis was made.

**Figure 5:**
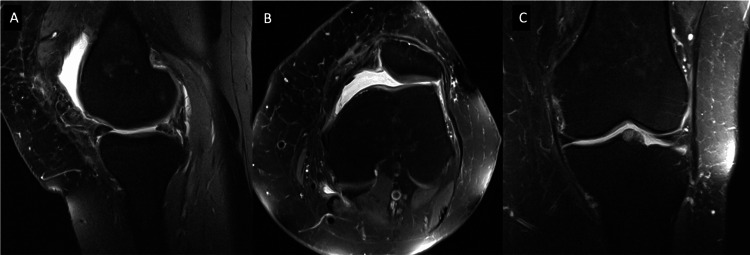
Post-operative MRI image of the knee PD FS MRI sequence: (A) sagittal, (B) axial, and (C) coronal views of the right knee demonstrating no evidence of lesion recurrence two years after surgical excision. PD, proton density; FS, fat suppression; MRI, magnetic resonance imaging

There were no complications in the immediate postoperative period or during follow-up. At two years, functional outcome was assessed: the patient had full extension and 120° of flexion, without instability, locking, or catching. She reported no limitations in daily activities and only occasional mild pain after prolonged walking. Follow-up MRI showed no evidence of recurrence (Figure 5).

Written informed consent was obtained from the patient for publication of this case report, including the clinical data and accompanying radiologic and intraoperative images. 

## Discussion

Although lipomas are the most common benign soft tissue tumors, representing almost half of all cases [1], true intra-articular lipomas are exceedingly rare. Approximately 27 cases have been reported in the literature, with the majority involving the knee joint and a few described in the hip, lumbar spine, and tarsometatarsal joints [2,3,4,12]. In a large series of 2,200 arthroscopic knee procedures, Özalay et al. identified only one lipomatous lesion, further underscoring the rarity of this condition [13].

Lipoma arborescens, one of the rarest synovial proliferative lesions, is characterized by fatty infiltration of the subsynovial connective tissue. It predominantly affects adult men and manifests as a slowly progressive swelling, most often involving the knee. Grossly, the synovium presents a bright yellow, villous, or nodular surface, distinct from the brown discoloration observed in pigmented villonodular synovitis or hemosiderotic synovitis. Microscopically, the subsynovial connective tissue is markedly thickened by mature adipose tissue, while the surface synovial cells appear reactive and epithelioid, without atypia or mitotic activity. Minor fatty infiltration within the subsynovial layer can be a physiological or degenerative finding, but the diagnosis of lipoma arborescens requires diffuse villous and nodular involvement of the synovium. It is generally regarded as a non-neoplastic process, and treatment by synovectomy is curative, with rare recurrence [14,15].

In contrast, a true intra-articular lipoma represents a solitary, encapsulated, and truly neoplastic lesion composed of mature adipocytes without synovial proliferation [11]. Reported cases span a broad age range, with a slight female predominance. Most patients present with progressive limitation of motion or mechanical symptoms, such as locking or patellar instability, secondary to the mass effect of the lesion [6]. Less frequently, acute pain and locking may result from strangulation or interposition of the mass between articular surfaces [7,8].

MRI remains the imaging modality of choice, confirming the fatty nature of the lesion and delineating its margins and relationship to intra-articular structures. True intra-articular lipomas appear as well-defined, homogeneous fat-containing masses, hyperintense on T1- and T2-weighted images and completely suppressed on FS sequences, sometimes with thin internal septations.

Surgical excision is curative, and both open and arthroscopic techniques have been reported. Arthroscopy offers a minimally invasive option for small and well-localized lesions, whereas larger or deeply situated masses may require open excision [11]. In our case, the lesion originated from the suprapatellar pouch and extended toward the medial aspect of the knee and distal thigh. Due to its considerable size and deep location, an open approach was selected, allowing complete resection without recurrence at follow-up.

## Conclusions

True intra-articular lipomas are rare lesions that typically have a slow and progressive onset. The main differential diagnosis is with other intra-articular lipomatous masses, particularly lipoma arborescens. MRI remains the imaging modality of choice, allowing accurate characterization of the lesion and differentiation between these entities. Regarding the treatment, excision may be done either by arthoroscopic or open approach depending on the size of the mass. The clinical course seems to be defined, especially by the location of the mass and its influence on the normal function of the joint. Recurrence is exceedingly rare in published cases, but given the scarcity of reports, conclusions cannot be generalized.

This case underscores the importance of correlating clinical presentation with imaging and histologic features to establish an accurate diagnosis. A multidisciplinary approach combining these findings is essential to distinguish true intra-articular lipoma from lipoma arborescens and guide appropriate management. Further research is warranted to clarify potential risk factors and to explore whether, and through which mechanisms, trauma-induced cytokine release and genetic predisposition may contribute to its pathogenesis.
